# Testosterone metabolites inhibit proliferation of castration- and therapy-resistant prostate cancer

**DOI:** 10.18632/oncotarget.24763

**Published:** 2018-03-30

**Authors:** Felix Bremmer, Hubertus Jarry, Valerie Unterkircher, Silke Kaulfuss, Peter Burfeind, Heinz-Joachim Radzun, Philipp Ströbel, Paul Thelen

**Affiliations:** ^1^ Institute of Pathology, University Medical Center, Göttingen 37075, Germany; ^2^ Department of Experimental Endocrinology, University Medical Center, Göttingen 37075, Germany; ^3^ Institute of Human Genetics, University Medical Center, Göttingen 37073, Germany; ^4^ Institute of Pathology, University Medical Center, Göttingen 37075, Germany; ^5^ Department of Urology, University Medical Center, Göttingen 37075, Germany

**Keywords:** castration resistant prostate cancer, AKR1C1, AKR1C2 and AKR1C3, 3a-androstendiol, 3b-androstendiol

## Abstract

Novel treatments for castration-resistant prostate cancer (CRPC) such as abiraterone acetate (AA) or enzalutamide effectively target the androgen pathway to arrest aberrant signalling and cell proliferation. Testosterone is able to inhibit tumour cell growth in CRPC. Estrogen receptor-beta (ERβ) binds the testosterone-metabolites 3β-androstanediol and 3α-androstanediol in parallel to the canonical estradiol. In the prostate it is widely accepted that ERβ regulates estrogen signalling, mediating anti-proliferative effects. We used the prostate cancer cell lines LNCaP, PC-3, VCaP, and the non-neoplastic BPH-1. VCaP cells were treated with 1 nmol/L testosterone over 20 passages, yielding the cell line VCaPrev, sensitive to hormone therapies. In contrast, LNCaP cells were grown for more than 100 passages yielding a high passage therapy resistant cell line (_hiP_LNCaP). VCaP and _hiP_LNCaP cell lines were treated with 5 μmol/L AA for more than 20 passages, respectively, generating the AA-tolerant-subtypes VCaP^AA^ and hiPLNCaP^AA^. Cell lines were treated with testosterone, dihydrotestosterone (DHT), R1881, and the androgen-metabolites 3β-androstanediol and 3α-androstanediol. 3β-androstanediol or 3α-androstanediol significantly reduced proliferation in all cell lines except the BPH-1 and androgen receptor-negative PC-3 and markedly downregulated AR and estrogen receptor alpha (ERα). Whereas ERβ expression was increased in all cell lines except BPH-1 or PC-3. In summary, 3β-adiol or 3α-adiol, as well as DHT and R1881, significantly reduced tumour cell growth in CRPC cells. Thus, these compounds represent novel potential therapeutic approaches to overcome drug-resistance in CRPC, especially with regard to AR-V7 function in therapy resistance. Furthermore, these data confirm the tumour suppressor properties of ERβ in CRPC.

## INTRODUCTION

Prostate cancer (PCa) is the most prevalent cancer among men in Europe. Its incidence has increased continuously over the past decades [1, 2]. In some cases, localised PCa can be cured by radical prostatectomy, radiotherapy, and/or androgen deprivation therapy [3]. However, castration-resistant PCa (CRPC) remains incurable, even after the development of highly active agents such as abiraterone (an inhibitor of steroidogenesis) or enzalutamide (a second-generation androgen receptor antagonist). Eventually, approximately 20–40% of patients do not respond to these agents, based on prostate-specific antigen (PSA) -response (i.e. primary resistance) [4–7]. Thus CRPC depends on AR-signalling for progression [8].

In addition to such compounds that prevent androgen synthesis (i.e. abiraterone) or androgen receptor antagonists (i.e. Enzalutamid or Bicalutamid) [9], derivatives of androgen biosynthesis such as testosterone or 5α -dihydrotestosterone (5α -DHT) effectively reduce tumour growth in castration resistant VCaP cells [10]. Several observations have demonstrated the significance of intratumoural androgen synthesis after chemical or surgical castration. Genes that are essential for steroid hormone synthesis, such as *AKR1C1*, *AKR1C2*, or *AKR1C3* are upregulated in CRPC. AKR1C1 and AKR1C2 convert 5a-DHT to 3α-androstanediol (3α-adiol) and 3β-androstanediol (3β-adiol), whereas AKRC1C3 converts Δ^4^-androstenedione to testosterone (Supplementary Figure 1) [9, 11].

Both testosterone-derivatives (3α-adiol and 3β-adiol) have significant affinity for estrogen receptor alpha (ERa) and beta (ERβ) [12, 13]. ERa is predominantly expressed in the prostate stroma and thus has a paracrine effect, whereas ERβ is expressed in the prostate epithelium and is decreased in PCa. ERβ protects against aberrant cell proliferation and carcinogenesis and thus is termed a tumour suppressor [14–18].

Another driver of CRPC is the overexpression of the androgen receptor (AR). Overexpression of AR compensates for reduced androgen levels, augmenting continuous tumour growth. This leads to a significant increase in AR-mediated gene transcription, including upregulation of *TMPSSR-ERG*, *IGF1*, and *PSA*, [9, 10]. Further molecular mechanisms include AR-splice variants such as ARV7, which constitutively activate AR [17–19]. Antonarakis *et al.* (2014) demonstrated an association between ARV7 and resistance to both enzalutamide and abiraterone in CRPC [20].

The abilities to overcome such therapy resistance by androgen metabolites such as 3α-adiol and 3β-adiol, in CRPC, have not been considered so far. Recently, we demonstrated the ability of the ER-subtype selective compound 8βVE-2 to reset resistance to hormone therapies [21]. In this study, we investigated the effect of 3α-adiol, and 3β-adiol on various PCa cells with regard to gain of function AR-aberrations and alternative gene splicing exemplified by AR-splice variant 7 (AR-V7).

## RESULTS

### Reduced proliferation of AR-positive PCa cells after treatment with 3α-diol

**Figure 1:**
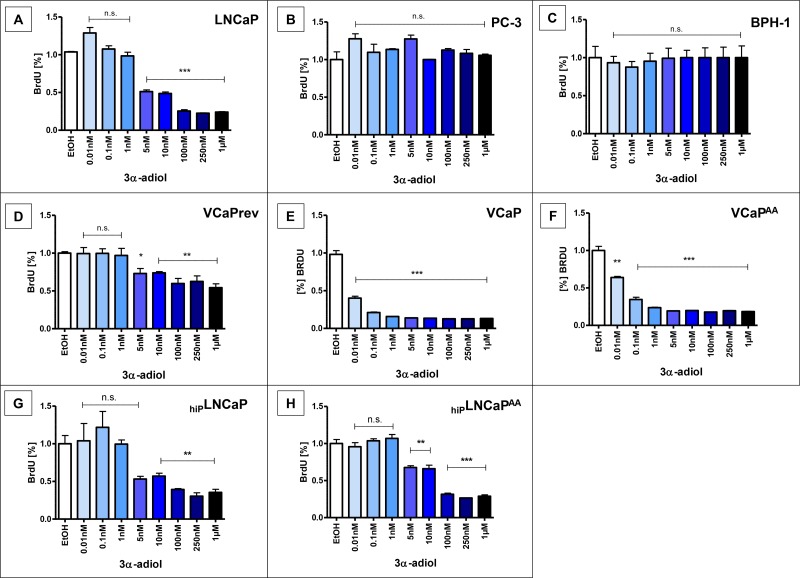
Proliferation after treatment with 3α-adiol Several prostate cancer cell lines were treated with 0, 0.01, 0.1, 1, 5, 10, 100, 250, or 1000 nmol/L 3α-adiol. Proliferation was assessed by performing BrdU-ELISA. 3α-adiol led to a significant reduction in proliferation in various LNCaP- and VCaP-derived cell lines, whereas androgen receptor (AR)-negative PC-3 and non-neoplastic BPH-1 cells showed no changes in proliferation (n.s. = not significant, ^*^P < 0.05, ^**^P < 0.005, ^***^P < 0.0005).

After 48 h treatment with 5 nmol/L to 1 μmol/L 3α-adiol, proliferation of LNCaP cells, was significantly reduced (*p* < 0.0005, Figure 1A), despite the gain-of-function AR mutation T878A, vastly defying hormone therapies. Such inhibition of proliferation with 3α-adiol treatments was not observed in AR-negative PC-3 or non-malignant BPH-1 cells (Figure 1B, 1C). In contrast, androgen-sensitive VCaPrev cancer cells represent a pre-androgen deprivation (preADT) therapy stage (Figure 1D). These cells also showed a significant reduction in proliferation with 5 nmol/L (*p* < 0.05) and 10 nmol/L to 1 μmol/L (*p* < 0.005) 3α-adiol; however, the effect was less prominent as compared with VCaP cells representing established CRPC and VCaP AA cells (Figure 1E, 1F). In addition, a significant reduction in proliferation was observed in the high passage cell line _hiP_LNCaP, albeit only with higher concentrations of 5 nmol/L to 1 μmol/L (*p* < 0.005), (Figure 1G) but basically unaltered from the original LNCaP (Figure 1A). In the same range of concentrations, 3α-adiol still significantly reduced proliferation in the therapy-resistant cell model _hip_LNCaP^AA^ (Figure 1H).

### Proliferation of prostate cells after treatment with 3β-adiol for comparison

**Figure 2:**
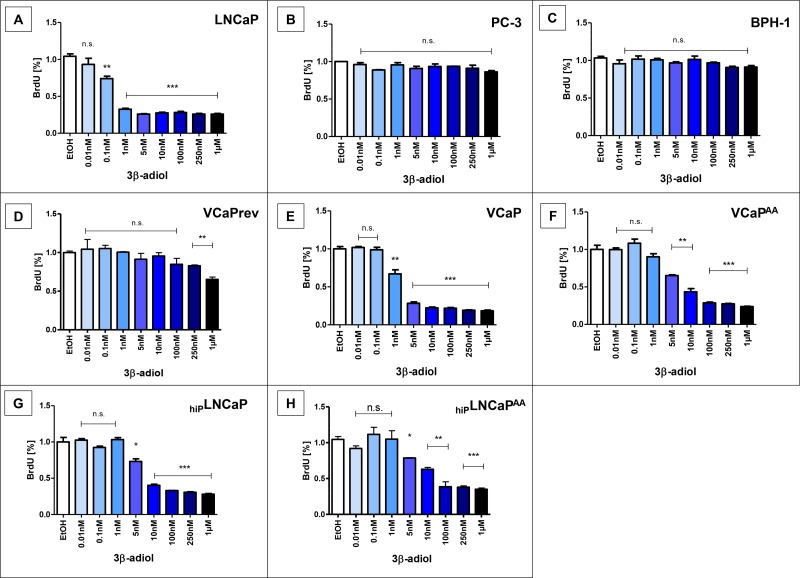
Proliferation after application of 3β-adiol Several prostate cancer cell lines were treated with 0, 0.01, 0.1, 1, 5, 10, 100, 250, or 1 nmol/L 3α-adiol. Proliferation was assessed by performing BrdU-ELISA. 3β-adiol led to a significant reduction in proliferation in LNCaP and VCaP derivatives, whereas PC-3 and BPH-1 showed no changes in proliferation (n.s. = not significant, ^*^P < 0.05, ^**^P < 0.005, ^***^P < 0.0005).

Effects on tumour cell proliferation of various CRPC models observed with 3α-adiol were also observed with 3β-adiol (Figure 2). In almost congruent concentration ranges the 3β-isoform of adiol also elicited decreased proliferation of PCa-cells with AR-aberrations but not in AR-negative PC-3 tumour cells (Figure 2B) and non-neoplastic AR-positive BPH-1 cells (Figure 2C). Not as effective as 3α-adiol in very low concentrations, 3β-adiol also shows the striking difference of adiol therapy potential between permanent CRPC, based on AR-gain-of-function models (LNCaP, Figure 2A, 2G, 2H) and CRPC, based on AR-deregulation and alternative AR-splicing (VCaP, Figure 2D–2F). Gain-of-function-AR LNCaP show decreased proliferation upon adiol treatments only above 1nM concentrations (Figures 1A, 1G and 1H, 2G–2H). A lower therapy potential is also obvious in VCaP cells before androgen deprivation, i.e. in the presence of 1 nM external testosterone (Figures 1D, 2D). However, with increasing ADT, first deprivation of external androgen (Figures 1E, 2E) and AA-treatment in addition (Figures 1F, 2F), there is an obvious potential to overcome CRPC from adiol treatments. For the therapy resistance mechanisms from ADT lowest adiol concentrations are sufficient to reduce tumour cell proliferation significantly with a slight advantage for 3α-adiol (Figure 1E–1F, Figure 2E–2F). In contrast, LNCaP-derived CRPC models with already permanent AR-mutations largely remain unaltered in their susceptibility to adiol treatments with increasing passage numbers (Figures 1 and 2; A compared with G) and AA-treatments (Figures 1 and 2; G compared with H).

### 3α-adiol or 3β-adiol arrest cell cycle in PCa cell lines

**Figure 3:**
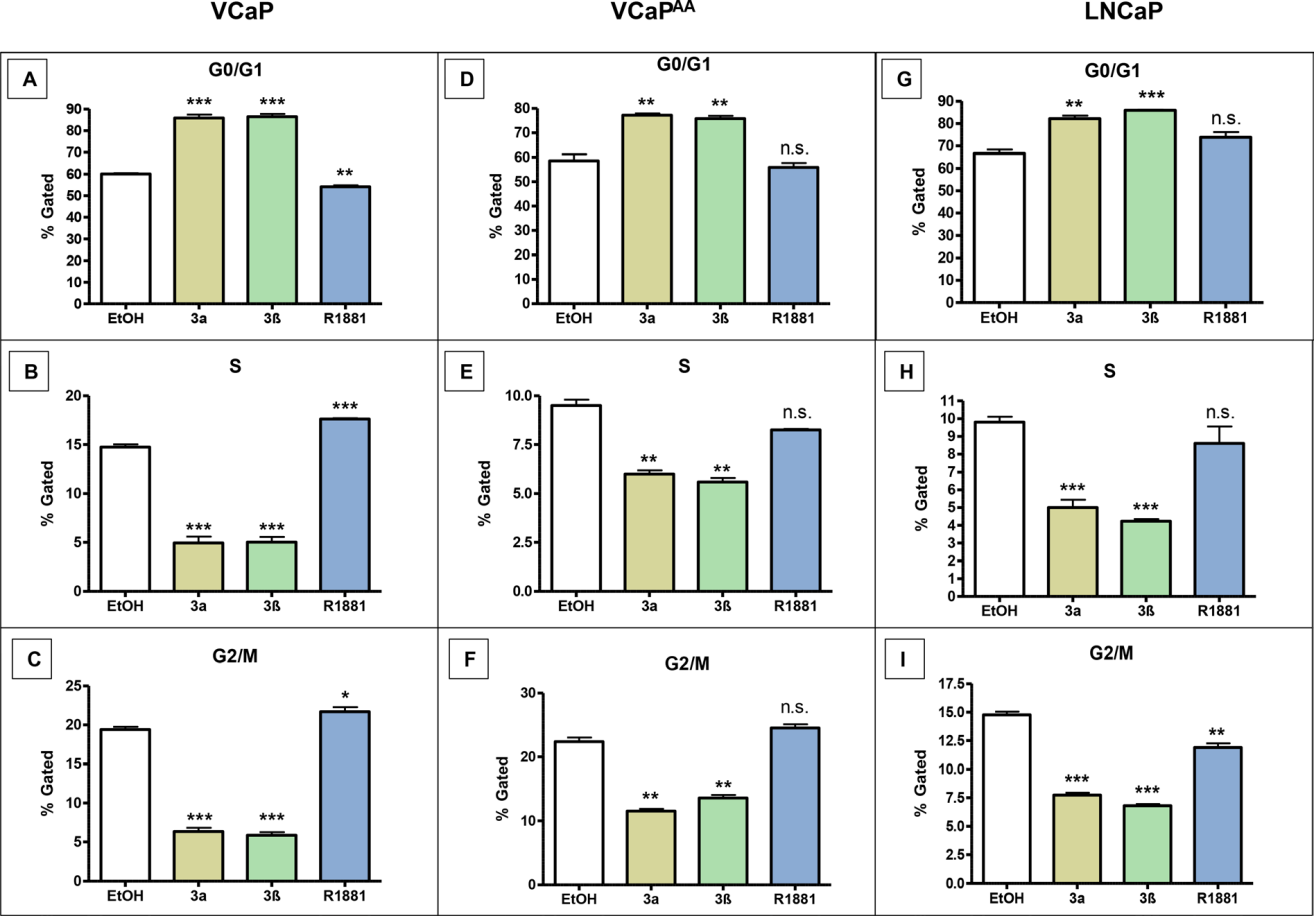
Cell cycle changes in various CRPC-cells after the application of 3α-adiol, 3β-adiol, and R1881 Prostate cancer cell lines were treated with 1 μmol/L 3α-adiol, 1 μmol/L 3β-adiol or 10 nmol/L R1881. Cell cycle was assessed using the commercial Muse™ Cell Cycle assay kit. In VCaP and VCaPAA cells, 3a-, 3β-adiol significantly increased cell numbers in the G0/G1 phases, whereas cells in the S or G2/M phases were significantly reduced. R1881 showed an opposite effect with a reduced number of cells in the G0/G1 phases and increased number of cells in the S and G2/M phases. In VCapAA cells, no significant changes were detected (**A**–**F**). In LNCAP 3a- and 3β-adiol application led to increases in cells in the G0/G1 phases, whereas the number of cells in S and G2/M phases was significantly reduced. R1881 resulted in inconsistent changes (**G**–**I**) (n.s. = not significant, ^*^P < 0.05, ^**^P < 0.005, ^***^P < 0.0005).

The effect of both adiols on PCa proliferation caused us to investigate cell cycle events. PCa cell lines were treated with 1 μmol/L 3α-adiol or 1 μmol/L 3β-adiol, or androgen stimulated with 10 nmol/L R1881 (Figure 3). Decreased tumour cell proliferation upon adiol treatments coincide with cells arrested in G0/G1 phase and less continuation to S- and G2/M phase. In comparison, androgen stimulation with R1881 does not show these effects on CRPC cell cycle in VCaP cells (data not shown). These cell cycle events are most pronounced for CRPC cells (Figure 3A–3C) and are still obvious after AA treatments (Figure 3D–3F), albeit untreated pre-ADT cells do not reveal cell cycle alterations from adiols or androgen R1881 (data not shown). In contrast G0/G1 arrest from the adiols but not from R1881 is obvious in all LNCaP cells (Figure 3G–3I) and its derivatives (data not shown).

This obvious growth constraint and cell cycle arrest induced in prostate cancer by adiols caused us to confirm the functional expression of the enzymes they originate from, especially AKR1C1 and AKR1C2. AKR1C1, AKR1C2 as well AKR1C3 are expressed in the presence of substrate androgens (VCaP rev and LNCaP with testosterone or DHT supplementation). AKR1C1 is slightly downregulated in the presence of its product 3β-adiol but no such effects became obvious for AKR1C2 and 3α-adiol (Supplementary Figure 2).

### Influence from adiol treatments on AR-, ERa-, and ERβ-expression

Since therapy resistance of CRPC is predominantly affected by AR characteristics (overexpression, gain-of-function mutations), we investigated AR expression in these different cell models and compared adiol treatments to androgen stimulation with non-metabolizable androgen R1881. Furthermore, we evaluated estrogen receptors (ERα and ERβ) as potential target for these compounds. PCa cell lines were treated with adiol concentrations of 1 μmol/L, an effective dosis in previous experiments, or 10 nmol/L R1881, which is comparable to physiological androgen concentrations. After treatment, western blotting was performed to investigate the expression of AR, ERα, and ERβ.

### VCaPrev, VCaP, and VCaP^AA^ cells (Figure 4A–4C)

**Figure 4:**
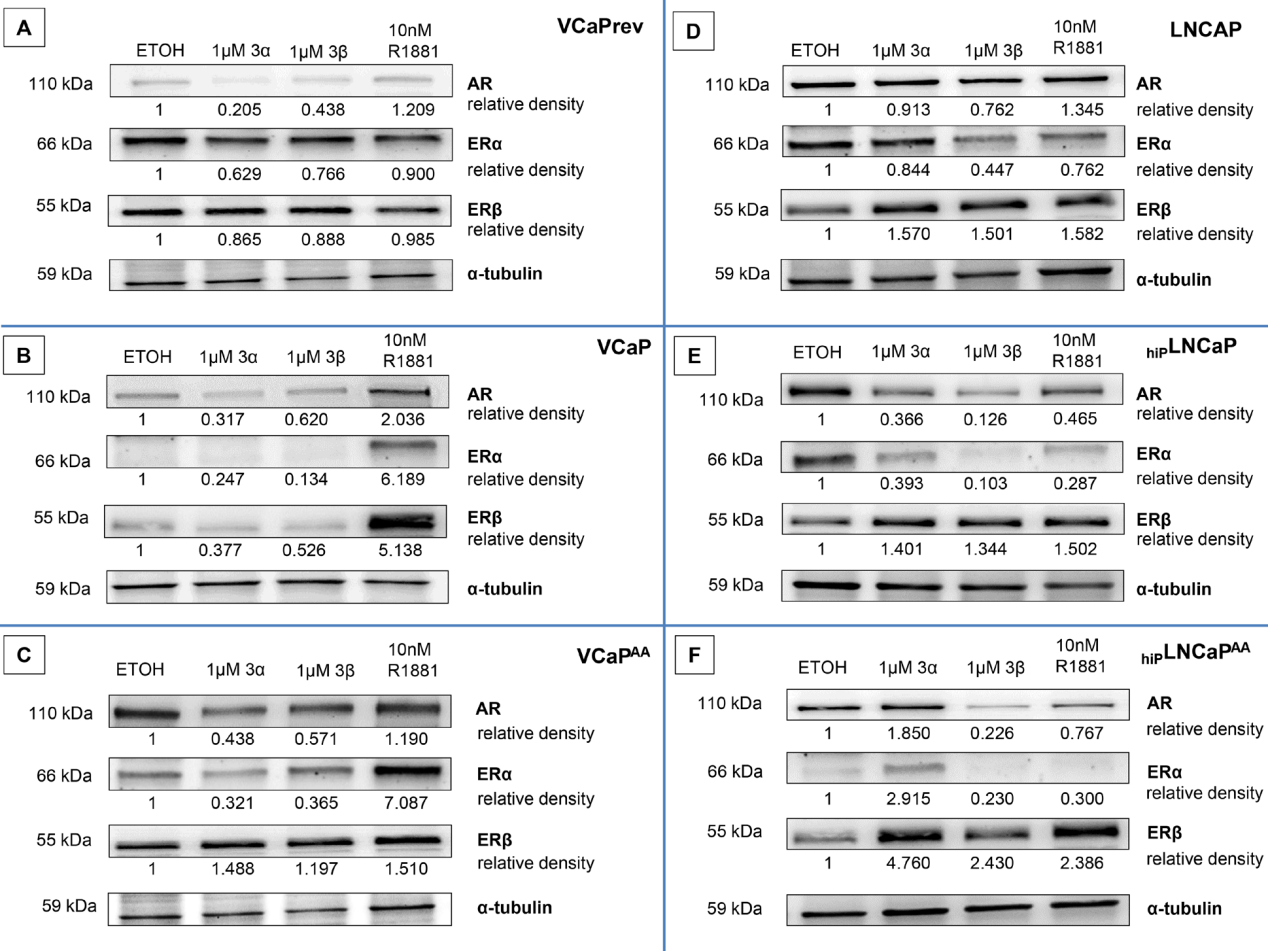
Androgen receptor (AR), Oestrogen receptor (ER)a, and ERβ expression after treatment with 3α-adiol, 3β-adiol, and R1881 Western blotting was performed to determine protein expression. 3α-adiol (1 μmol/L) or 3β-adiol (1 μmol) considerably reduced AR expression in VCaPrev cells, whereas 10 nmol/L R1881 resulted in a slight induction of AR expression. In addition, no significant changes in ERa or ERβ expression were detectable (**A**). In VCaP cells, 1 μmol/L 3α-adiol or 1 μmol/L 3β-adiol reduced AR expression, whereas 10 nmol/L R1881 resulted in elevated AR expression. R1881, but not 3α-adiol and 3β-adiol, stimulated the expression of ERa or ERβ (**B**). 3α-adiol (1 μmol/L) or 3β-adiol (1 μmol/L) markedly reduced AR expression in VCaPAA cells, whereas 10 nmol/L R1881 resulted in a slight increase in AR expression. 3α-adiol (1 μmol/L) or 3β-adiol (1 μmol/L), as well as androgen stimulation with R1881, reduced ERa expression and increased ERβ expression (**C**). The opposite regulation of protein expression (downregulation for ERa and upregulation for ERβ) by 1 μmol/L 3α-adiol or 1 μmol/L 3β-adiol was even more pronounced for LNCaP derivatives (**D**–**F**).

Pre-therapy androgen-sensitive VCaPrev moderately express AR in the presence of sufficient, but sub-physiological (1 nM, 0.3 ng/ml) testosterone. But also from this low expression level, 3α-adiol or 3β-adiol treatments led to a considerable reduction in AR expression, whereas the androgen R1881 increased AR expression. No such divergent effects from adiols and R1881 were observed on ERα and ERβ expression (Figure 4A). VCaP cells with no external testosterone in the cell culture represent castration resistant growth (Figure 4B). Under this CRPC condition the AR is slightly up-regulated as compared with the pre-ADT state (Figure 4A). In contrast to AR the estrogen receptors are down-regulated under CRPC condition and they are further down-regulated by adiol-treatments but up-regulated parallel to AR by a stimulatory treatment with R1881 (Figure 4B). The therapy-resistant VCaP derivative VCaP^AA^ cells showed the highest expression of AR. With increasing androgen deprivation, AR expression increased to levels significantly above those of VCaPrev and VCaP cells. However, treatment with 3α-adiol or 3β-adiol markedly reduced AR expression, whereas R1881 resulted in only a slight increase in AR expression. In addition, 3α-adiol or 3β-adiol also resulted in a considerable reduction in ERα expression. Interestingly, treatment with R1881 resulted in a dramatic increase in ERα. Finally, treatment with 3α-adiol, 3β-adiol, or R1881 markedly increased ERβ expression (Figure 4C).

### LNCaP, _hiP_LNCaP, and _hiP_LNCaP^AA^ cells (Figure 4D–4F)

LNCaP cells *per se* represent the T878A gain-of-function mutation and less susceptibility to ADT. To obtain cell models with increased potential for therapy resistance before this background, we established a high-passage LNCaP (_hiP_LNCaP) and AA-tolerant derivatives (_hiP_LNCaP^AA^).

There was also considerable expression of the steroid receptors AR, ERα, and ERβ in LNCaP CRPC cells. In the VCaP model, increasing resistance to androgen deprivation was evident by accumulating AR overexpression (Figure 4A–4C); however, there was no such development in the LNCaP model with the gain-of-function mutation T878A (Figure 4D–4F). Accordingly, treatment with 3α-adiol, 3β-adiol, or R1881 had no significant effect on AR expression in basal LNCaP cells. However, ERα expression was reduced after treatment with 3α-adiol or R1881, and markedly reduced with 3β-adiol. Interestingly, up-regulation was observed for ERβ upon 3α-adiol-, 3β-adiol-, or R1881-treatments (Figure 4D). Moreover, the therapy resistant derivatives from LNCaP, established by prolonged growth under androgen deprivation (Figure 4E) and AA-treatment (Figure 4F) illustrate decisively an ERβ acting as a counterbalance on AR- and ERα-expression. This antiandrogen action from ERβ was most pronounced after 3β-adiol-treatments (Figure 4F).

### Effect of 3α-adiol and 3β-adiol on ARV7 expression

**Figure 5:**
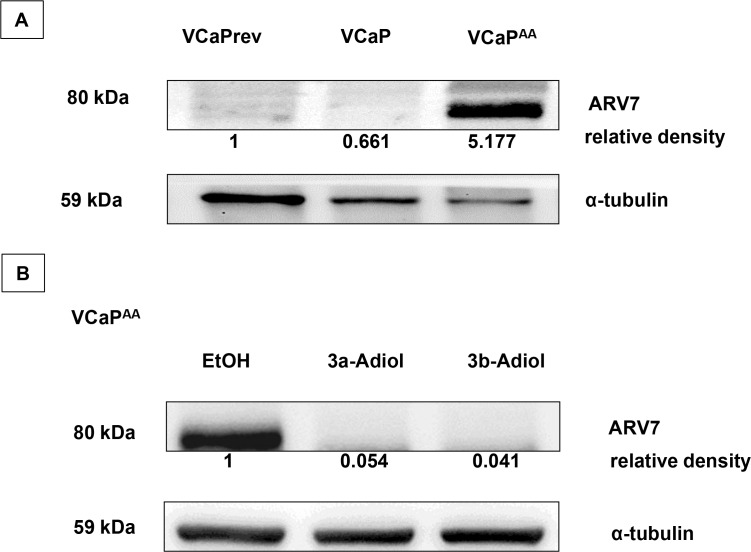
ARV7 expression in VCaP under increasing androgen deprivation With increasing androgen deprivation therapy (ADT), the expression of full-length androgen receptor (flAR) continuously increased (as shown in Figure 4A–4C), which was accompanied by a stepwise increase in specific antibody staining for the AR splice variant 7 (ARV7), but only with maximum ADT (**A**). The strong ARV7 signal in the presence of abiraterone acetate was reversible upon additional treatments of VCaP AA cells with 1 μmol/L 3α-adiol or 3β-adiol (**B**).

Recent clinical observations linked therapy resistance and cross resistance to expression of AR splice variants such as AR-V7. Therefore, we analysed the function of AR-V7 in therapy resistance in our cell models (Figure 5). As shown in Figure 4, increasing androgen deprivation and AA-treatment in the VCaP model resulted in accumulating overexpression of wild-type AR (EtOH-control experiments, Figure 4A–4C). This observation was reproduced in experiments with focus on AR-V7 with specific antibodies. In contrast to an evolving overexpression of AR full length under ADT, the appearance of AR-V7 is stepwise only after fulfilment of ADT with AA-treatments (Figure 5A). All experiments done with the CRPC model T878A-AR LNCaP showed negative results for AR-V7 or other AR splice-variants with N-terminal AR antibodies or specific AR-V7 antibodies (data not shown). As demonstrated in our experiments, the function of AR-overexpression in therapy resistance was reversible with adiol treatments. The appearance of AR-V7 in VCaP^AA^-cells was also reversible by adiol treatments with a slightly better efficacy from 3β-adiol (Figure 5B).

## DISCUSSION

Conventional anti-hormonal agents, used for the primary treatment of metastatic PCa, are intended to prevent AR activation and curb tumour cell proliferation. Thus, androgen deprivation with LHRH (or GnRH) agonists and antagonists (LHRHa) reduces testosterone production (primarily in the testes) and the number of AR ligands [9]. The efficacy of these compounds typically decreases due to the development of resistance, which undermines androgen deprivation therapy (ADT). Upon new tumour growth, even though serum testosterone levels mimic castration levels, PCa has reached the stage of castration-resistance [22]. Castration resistance can be achieved by two distinct mechanisms represented by the two cell models used in this study, specifically, VCaP (AR aberration) and LNCaP (gain-of-function). The VCaP model allows for androgen-independent growth via AR overexpression with low testosterone. Completely androgen independent growth is mediated by constitutive active AR splice variants (Figure 5). The LNCaP model permits growth in the absence of androgen, due to the promiscuous use of substitute androgens such as estradiol or flutamide. Therefore, in case of castration resistance, for metastatic PCa, the prognosis is usually dismal.

New AR targeting therapies have been developed for the treatment of patients with VCaP based on evidence emphasizing the importance of AR signalling. Therefore, it is generally accepted that AR signalling is a key driver of CRPC [23, 24].

Novel treatments such as AA, which suppresses ligand synthesis, or enzalutamide, which completely blocks the AR, effectively target the androgen pathway to arrest aberrant signalling, and are therefore most suitable for CRPC treatment [5–7, 25]. However, both functional agents can fail due to AR splice variants [20]. AR overexpression and AR-V occurrence might be reversible but gain-of-function somatic mutations persist even with drug withdrawal. In this regard, it has become evident that androgens are able to eliminate tumour cell growth in CRPC (Supplementary Figures 3–5) [10]. The concept of bipolar androgen therapy has proven efficient in overcoming therapy resistance due to androgen exposure [26]. Especially in VCaP tumour cells, high levels of testosterone can induce DNA double strand breaks and disrupt the function of AR. This was shown to result in inhibition of proliferation in PCa cells [26].

These findings are similar to the results in our study. We confirmed that low (nmol/L) concentrations of R1881, testosterone, and 5a-DHT effectively reduce proliferation in LNCaP, VCaP, VCaP^AA^, _hiP_LNCaP, and _hiP_LNCaP^AA^ cells, whereas the original pre-treatment cell model VCaPrev only showed marginal androgen-stimulated proliferation. Furthermore, it is evident that intratumoural steroidogenesis is crucial in CRPC. In addition, it is known that the androgen biosynthesis enzymes AKR1C1, AKR1C2, and AKR1C3 are upregulated in prostate cancer [9]. AKR1C1 and AKR1C2 convert 5a-DHT to 3α-adiol and 3β-adiol [11]. However, the expression of AKR1C1, AKR1C2, and AKR1C3 did not significantly differ after treatment with 3α-adiol or 3β-adiol (Supplementary Figure 2).

Notably, we did show that 3α-adiol or 3β-adiol leads to a significantly reduced proliferation in LNCaP, VCaPrev, VCaP, VCaP^AA^, _hiP_LNCaP, and _hiP_LNCaP^AA^ cells, whereas 3α-adiol or 3β-adiol did not affect BPH-1 and PC-3 cell proliferation. Guerini *et al.* (2005) demonstrated that 3α-adiol and 3β-adiol reduces tumour cell migration in AR-negative DU145 PCa cells. Inhibition of DU145 cell motility has been associated with the formation of the testosterone/dihydrotestosterone metabolite 3β-adiol, which acts through the activation of ERβ, which is present in these PCa cells [27]. In the prostate, ERβ has features of a tumour suppressor. It is widely accepted that ERβ adopts a regulatory role in estrogen signalling, mediating anti-proliferative effects. In this study, we investigated the effects of testosterone-metabolites with regard to proliferation and estrogen signalling.

In addition, it has been shown that 3α-adiol and 3β-adiol, two metabolites that do not bind the AR, have a higher affinity for estrogen receptors [13, 28, 29]. We therefore investigated the expression of ERα, ERβ, and AR in VCaP cells after treatment with 1 μmol/L 3α- and 3β-adiol. Interestingly, this led to reduced AR expression, whereas ERα and ERβ were relatively unchanged. In addition, in gain-of-function AR-bearing LNCaP cells, AR expression was reduced after treatment with 1 μmol/L 3α- and 3β-adiol. In these cells, the expression of ERα was also reduced, whereas ERβ protein expression was considerably increased. We hypothesized, according to our previous studies, that 3β-adiol binds/activates ERβ, leading to downregulation of the AR [14]. Because the goal of CRPC therapy is to prevent activation of the AR, 3α- and 3β-adiol could be potential therapeutic options for CPRC treatment, without targeting the AR directly. Therefore, we conclude that these drugs have universal therapeutic potential, independent of CRPC characteristics and the promiscuous utilisation of steroids (Figures 4 and 5).

We further demonstrated that the decrease in proliferation after treatment with 3β-adiol or 3α-adiol could be explained by a significant increase in tumour cell cycle arrest. This decrease is in agreement with results from Weihua *et al.* (2002), in which inhibition of growth in the developing ventral prostate was achieved with 3β-adiol [30].

Therapy resistance and cross resistance can be explained by constitutively active AR splice variants subverting further ADT by abiraterone, and by the loss of the specific targets for enzalutamide. The ERβ-mediated anti-androgen effect has more universal potential, via the downregulation of such androgen-independent functions, and most importantly for gain-of-function mutations. This functional reciprocal counteraction of ERβ activation and AR suppression was most prominent for 3β-adiol in therapy-resistant _hiP_LNCaP^AA^ cells (Figure 4F) but was also evident for other LNCaP models (Figure 4D, 4F). Counteraction of ERβ on ERα was also evident (Figure 4D–4F) [31, 32]. We recently investigated this reciprocal mechanism with the ERβ subtype-selective drug 8ßVE-2 [21]. Furthermore, we investigated the expression profile of AKRs with therapy resistance by investigating AR splice variants (Supplementary Figure 2). The occurrence of ARV7 appeared to re-introduce the profile of intact androgen signalling in an androgen-free environment (Figure 5). 3β-adiol is synthesized from 5α-DHT by AKR1C1, which is highly overexpressed with positive expression of ARV7 (Figure 5A and Supplementary Figure 2). This phenomenon might be explained by a feedback mechanism from complete AA-induced ADT and the established absence of DHT. However, this was reversed with experimental supplementation with 3β-adiol (Figure 5B).

In conclusion, our data suggest that the testosterone derivatives 3α-adiol and 3β-adiol are able to inhibit tumour cell growth in several PCa cell lines and this decrease in proliferation could be explained by cell cycle arrest. ERα and ERβ, as potential receptors of 3α-adiol and 3β-adiol, seem to play an important role in PCa therapy resistance, potentially by influencing AR deregulation. Since these compounds evoked a decrease in AR expression, they could represent a potential therapeutic option, especially when therapy resistance by AR-aberrations occur in CRPC.

## MATERIALS AND METHODS

### Culture of PCa cell lines

We used the human prostate cancer cell lines VCaP (LGC Standards Teddington, England), and LNCaP, BPH-1 and PC-3 from local certified stocks as previously described [33, 34]. Briefly, for experiments cells were kept under 35 passages in phenol red-free Gibco^®^ DMEM lot # 1089200 (Life Technologies GmbH, Darmstadt, Germany) supplemented only with 2% sodium pyruvate and 10% fetal bovine serum, steroid-free according to the manufacturer´s data sheet (PAA, Cölbe, Germany). To establish an androgen dependent variant VCaP cells were treated with 1 nmol/L testosterone (Sigma-Aldrich, Taufkirchen, Germany) over 20 passages yielding the androgen-sensitive cell line (VCAPrev) and for the generation of a therapy resistant cell line (VCaP^AA^) VCaP cells were treated with 5 μmol/L AA (Janssen Cilag, Neuss, Germany) over 20 passages [21]. To demonstrate enduring resistance to prolonged androgen deprivation, also a high passage (>100) variant from the gain-of-function AR cell line LNCaP was used (_hiP_LNCaP) [32], and was treated with 5 μmol/L AA (_hiP_LNCaP^AA^). Cells were incubated at 37°C and 5% CO_2_ in a humidified incubator. Cells and supernatants were harvested for protein extraction or used for ELISA or FACS assays.

### Assessment of cell proliferation

Tumour cell proliferation was evaluated by BrdU-ELISA. Cells were treated with 0.1, 1, or 10 nmol/L testosterone (article number: T6147, Sigma-Aldrich, Taufkirchen, Germany); 0.1, 1, 5, or 10 nmol/L DHT (article number: D5027, Sigma-Aldrich, Taufkirchen, Germany) and R1881 (article number: R0908, Sigma-Aldrich, Taufkirchen, Germany); and 0.01, 0.1, 1, 5, 10, 100, 250, or 1000 nmol/L 3α-adiol and 3β-adiol (article number: A1170-000 for 3α-adiol and A1220-000 for 3β-adiol, both from Steraloids, Newport, Rhode Island, USA) for 48 h.

### Western blotting

Tumour cells were treated with 1 μmol/L 3α-adiol, 1 μmol/L 3β-adiol, 10 nmol/L R1881T, 10 nmol/L testosterone, or 10 nmol/L DHT. Total protein lysates were prepared using RIPA buffer with protease inhibitors (Roche, Germany). Concentrations were quantified by Bio-Rad DC Protein Assays (Bio-Rad, USA). Primary antibodies are listed in Table 1. Antibodies were detected by polyclonal rabbit anti-mouse immunoglobulins/HRP (1:1000, Dako, DK). Membranes were developed using ECL (Amersham Bioscience, Germany).

**Table 1:** Antibodies used in this study

Antibody	Clone/article number	host	Company	Solution	kDa
androgen receptor (AR)	Ab-2	rabbit polyclonal	ThermoFisher	1:200	110
estrogen receptor alpha (ERa)	MC-20	rabbit polyclonal	Santa Cruz	1:1000	66
estrogen receptor beta (ERβ)	4D2	mouse monoclonal	GeneTex	1:500	58–60
aldo-keto reductase family 1 member C1 (AKR1C1)	859026	mouse monoclonal	R&D systems	1:1000	37
aldo-keto reductase family 1 member C2 (AKR1C2)	13035	rabbit polyclonal	Cell signalling	1:1000	37
aldo-keto reductase family 1 member C3 (AKR1C3)	871701	mouse monoclonal	R&D systems	1:1000	45
androgen receptor splice variant-7 (ARV7)	EPR15656	rabbit monoclonal	abcam	1:1000	80
α-tubulin	11H10	rabbit monoclonal	Cell signalling	1:1000	52

### Assessment of cell cycle

Cell cycle was evaluated after treatment with 1 μmol/L 3α-adiol, 1 μmol/L 3β-adiol, or 10 nmol/L R1881 for 48 h, using the Muse™ Cell Cycle assay as described by the manufacturer. Treated tumour cells (200 μL) were centrifuged at 300 × *g* for 5 min and washed with PBS; 200 μL of ice-cold 70% ethanol was then added. Subsequently, cells were incubated for 3 h at −20°C and 200 μL of Muse^™^ Cell Cycle Reagent were added. Finally, tumour cells were incubated for 30 min at room temperature in the dark. Muse Cell Cycle Software Module was used for automatic calculations.

### Statistical analysis

The mean ± SD and *P* values were evaluated with GraphPad Prism software version 5.0 using an unpaired non-parametric *t* test and a 95% confidence interval (n.s. = not significant, ^*^p < 0.05, ^**^p < 0.005, and ^***^p < 0.0005).

## SUPPLEMENTARY MATERIALS FIGURES


