# Supplementary material for: Testosterone metabolites inhibit proliferation of castration- and therapy-resistant prostate cancer
Source: Oncotarget. 2018 Mar 30;9(24):16951–61. doi: 10.18632/oncotarget.24763 (PMC5908297; doi:10.18632/oncotarget.24763)
Supplement: Supplementary file 1 [file oncotarget-09-16951-s001.pdf]

Testosterone metabolites inhibit proliferation of castration- and therapy-resistant prostate cancer

SUPPLEMENTARY MATERIALS

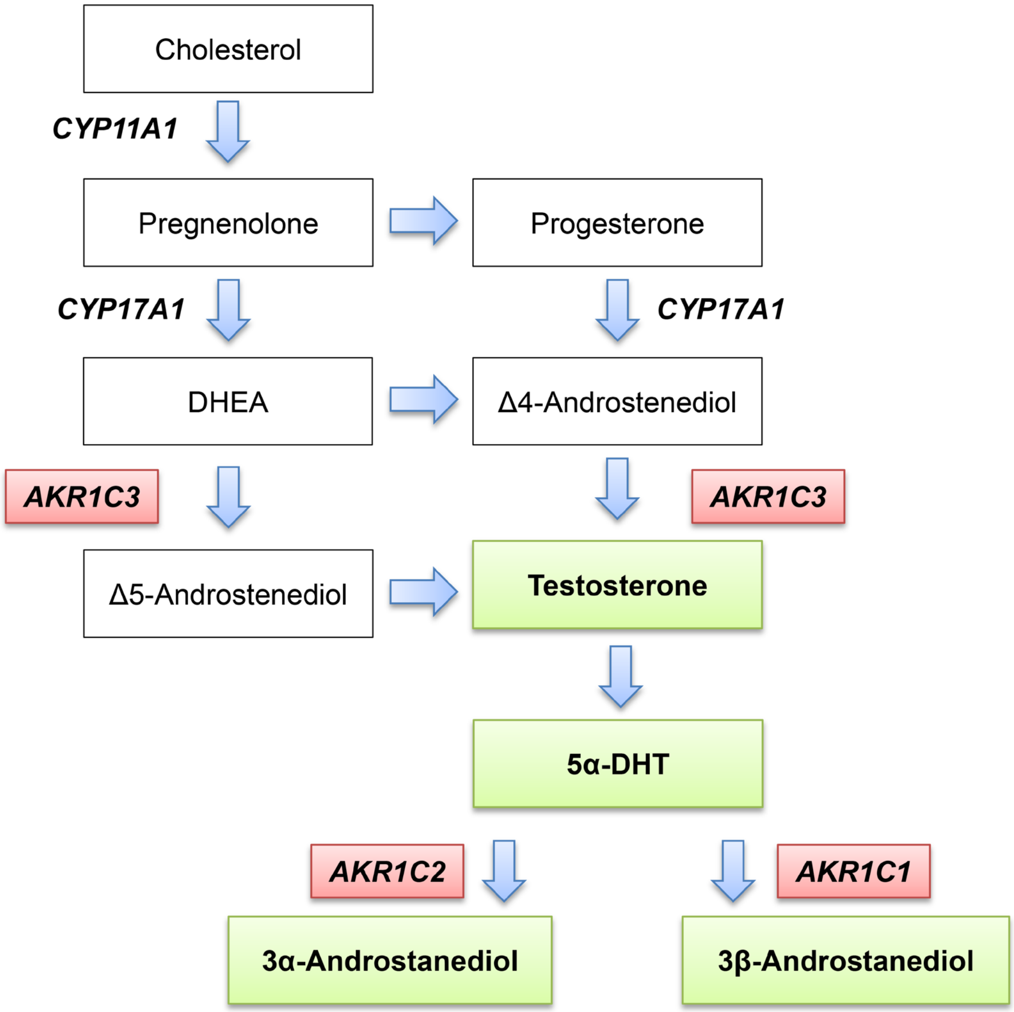

Supplementary Figure 1: Steroidogenesis in prostate cancer: Androgen receptor (AR) affinity peaks in DHT by 5α-reductase conversion of testosterone. 3β-adiol or 3α-adiol (3β-androstanediol, 3α-androstanediol) are metabolites of 5α-DHT, converted by AKR1C1 (3β-adiol) or AKR1C2 (3α-adiol) with reduced AR-affinity but increased estrogen receptor affinity. Modified from Knudsen and Penning [9].

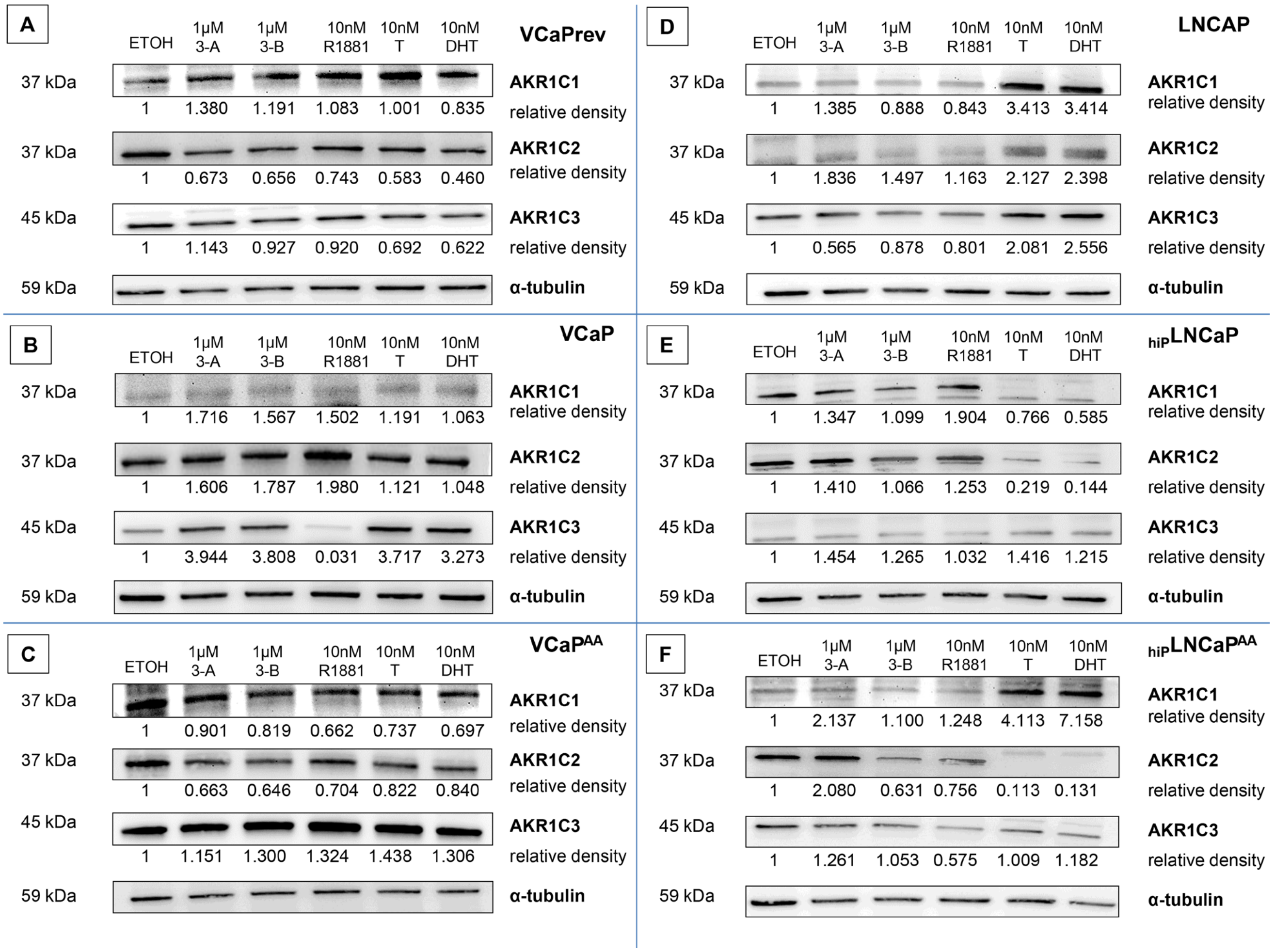

Supplementary Figure 2: Expression of AKR1C1, AKR1C2, and AKR1C3 in prostate cancer cell lines. In VCaPrev cells, 1 µmol/L 3α-adiol, 1 µmol/L 3β-adiol, 10 nmol/L R1881, 10 nmol/L testosterone, and 10 nmol/L 5a-DHT resulted in no marked changes in AKR1C1 and AKR1C3 expression, whereas AKR1C2 expression was markedly reduced (**A**). In VCaP cells, AKR1C1, AKR1C2, and AKR1C3 expression was upregulated after application of 1 µmol/L 3α-adiol, 1 µmol/L 3β-adiol, 10 nmol/L testosterone, and 10 nmol/L 5a-DHT, with the following exception; R1881 had not effect on AKR1C3 (**B**). VCaPAA expressed AKR1C1, AKR1C2, and AKR1C3. Treatment with 1 µmol/L 3α-adiol, 1 µmol/L 3β-adiol, 10 nmol/L R1881, 10 nmol/L testosterone, and 10 nmol/L 5a-DHT effects downregulation of AKR1C1 or AKR1C2 expression and upregulation of AKR1C3 (**C**). Low expression of AKR1C1 and AKR1C2 and moderate expression of AKR1C3 was observed in LNCaP cells. 3α-adiol (1 µmol/L), 3β-adiol (1 µmol/L), and R1181 had various moderate effects on the basal expression of these enzymes, whereas treatment with 10 nmol/L testosterone and 10 nmol/L 5a-DHT considerably increased AKR1C1, AKR1C2, and AKR1C3 expression (**D**). In addition, the application of 1 µmol/L 3α-adiol, 1 µmol/L 3β-adiol, or 10 nmol/L R1881 resulted in no changes in the expression of AKR1C1, AKR1C2, and AKR1C3. In contrast to that in LNCaP cells, treatment with 10 nmol/L testosterone and 10 nmol/L 5a-DHT considerably increased the expression of AKR1C1 and AKR1C2 in hiPLNCaP cells. Not all compounds affected AKR1C3 expression (**E**). In abiraterone acetate-treated hiPLNCaP cells, the most significant effects were from testosterone and 5a-DHT, in terms of AKR1C1 upregulation and AKR1C3 downregulation (**F**).

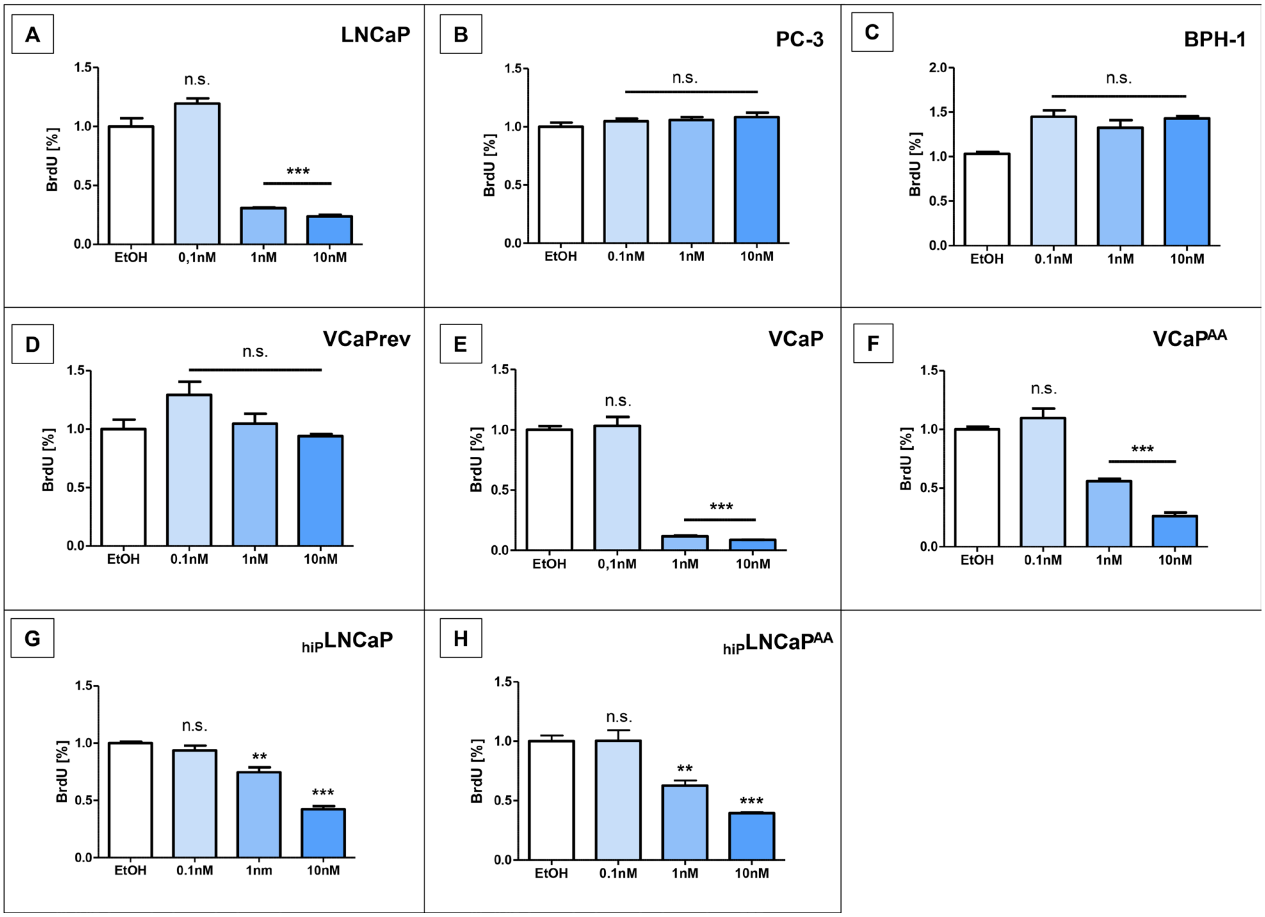

Supplementary Figure 3: Androgen stimulation in various cell types. In LNCaP, VCaP, VCaPAA, hiPLNCaP, and hiPLNCaPAA cells, proliferation, as assessed by BrdU-ELISA, was significantly reduced after treatment with 1–10 nmol/L testosterone (**A**, **E**–**H**). Testosterone resulted in no changes in proliferation for PC-3 cells and moderate effects for BPH-1 cells (**B** and **C**), and sustained proliferation in testosterone-sensitive prostate cancer cells (**D**) (n.s. = not significant, $^{*}P < 0.05$, $^{**}P < 0.005$, $^{***}P < 0.0005$).

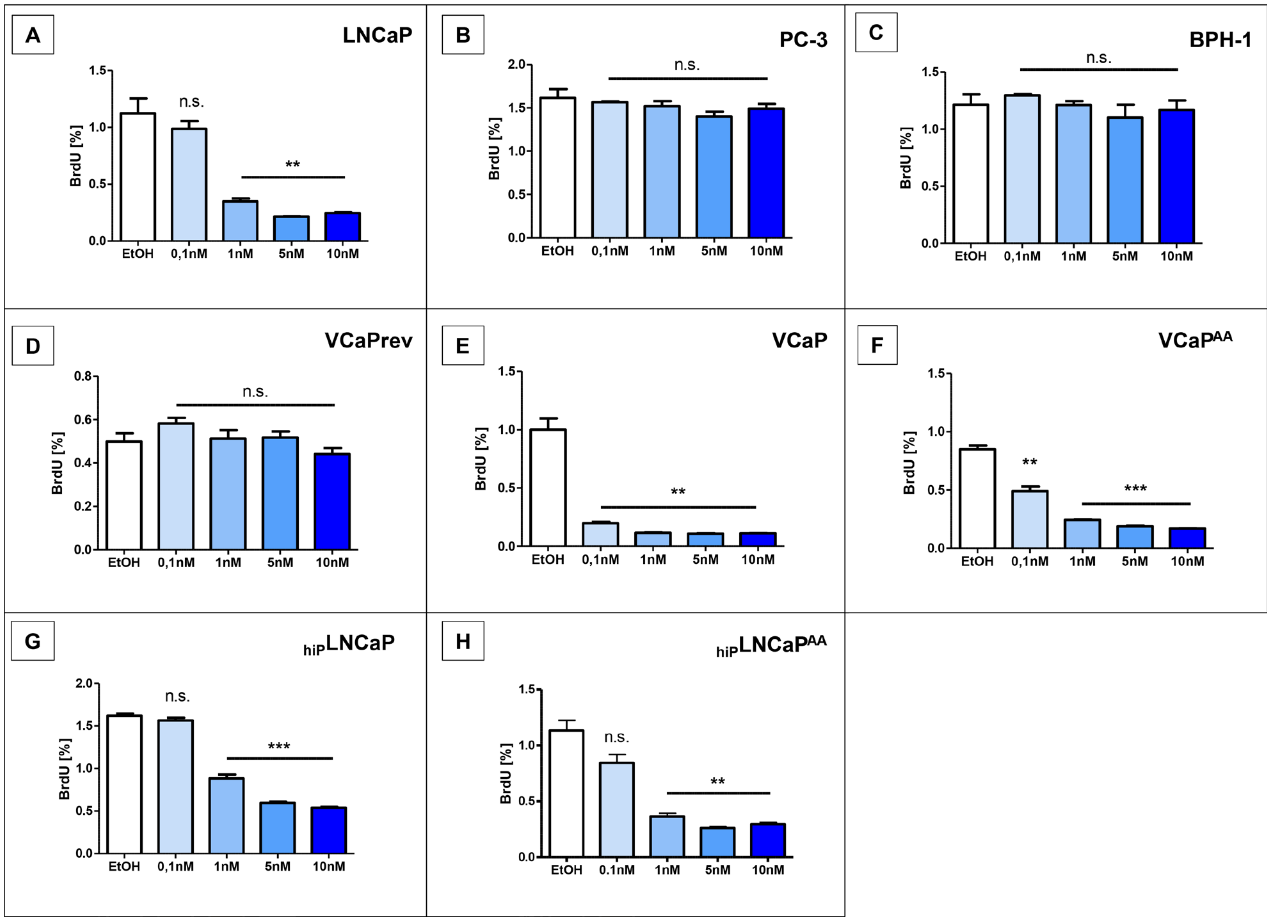

Supplementary Figure 4: Effects of 5a-DHT on various prostate cancer cells. Treatment with 1–10 nmol/L 5a-DHT in LNCaP led to a significant reduction in proliferation, as assessed by BrdU-ELISA; 0.1 nmol/L resulted in no reduction in proliferation in LNCaP cells (**A**). In PC-3, BPH-1, and VCaPrev cells, no changes in proliferation were detected (**B**–**D**). Treatment with 0.1 nmol/L, 1 nmol/L, 5 nmol/L, or 10 nmol/L 5a-DHT significantly reduced proliferation in VCaP and VCaPAA cells (**E** and **F**). Treatment of HiPLNCaPs resulted in similar effects as treatment of basal LNCaP (**G**, **H**) (n.s. = not significant, $^{*}P < 0.05$, $^{**}P < 0.005$, $^{***}P < 0.0005$).

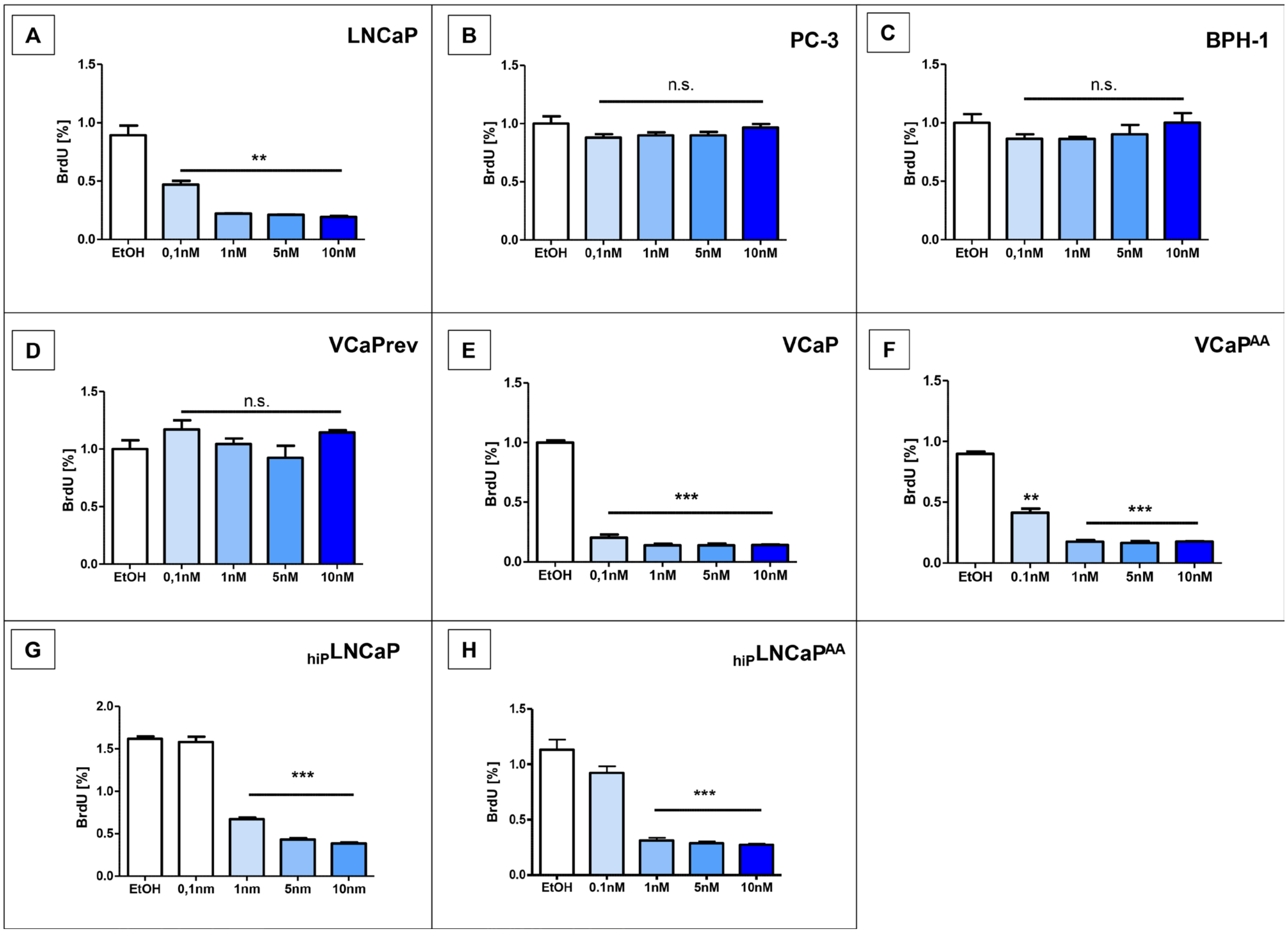

Supplementary Figure 5: Effects of the non-metabolisable androgen R1881 on various prostate cancer cells. PCa cells were treated with 0.1 nmol/L, 1 nmol/L, 5 nmol/L, and 10 nmol/L R1881. Proliferation of LNCaP, VCaP, VCaPAA, hiPLNCaP, and hiPLNCaPAA cells, as assessed by BrdU-ELISA, was comparable to that with testosterone and 5a-DHT (n.s. = not significant, $^{*}P < 0.05$, $^{**}P < 0.005$, $^{***}P < 0.0005$).
